# Treatment with PCSK9 Inhibitor Evolocumab Improves Vascular Oxidative Stress and Arterial Stiffness in Hypercholesterolemic Patients with High Cardiovascular Risk

**DOI:** 10.3390/antiox12030578

**Published:** 2023-02-25

**Authors:** Alessia Silla, Federica Fogacci, Angela Punzo, Silvana Hrelia, Patrizia Simoni, Cristiana Caliceti, Arrigo F. G. Cicero

**Affiliations:** 1 Department for Life Quality Studies, University of Bologna, 40126 Bologna, Italy; 2 Department of Medical and Surgical Sciences, University of Bologna, 40138 Bologna, Italy; 3 IRCCS Policlinico S. Orsola-Malpighi di Bologna, 40138 Bologna, Italy; 4 Department of Chemistry “Giacomo Ciamician”, University of Bologna, 40126 Bologna, Italy; 5 Department of Biomedical and Neuromotor Sciences—DIBINEM, University of Bologna, 40126 Bologna, Italy; 6 Istituto Nazionale Biosistemi e Biostrutture (INBB), 00136 Rome, Italy; 7 Interdepartmental Center of Industrial Research (CIRI)—Energy and Environment, Alma Mater Studiorum, University of Bologna, 40126 Bologna, Italy

**Keywords:** Evolocumab, PCSK9 inhibitor, lipid-lowering treatment, PBMC_S_, H_2_O_2_, chemiluminescence, arterial stiffness, carotid–femoral pulse wave velocity

## Abstract

Atherosclerosis and atherosclerotic-related cardiovascular diseases (ASCVD) are characterized by high serum levels of low-density lipoprotein cholesterol (LDL-C) that can promote the generation of reactive oxygen species (ROS). To answer the need for better LDL-C control in individuals at high and very high risk for CVD, a new injectable innovative family of lipid-lowering (LL) monoclonal antibodies against the protein convertase subtilisin/kexin type 9 (PCSK9) has been approved. However, the effect of these drugs on vascular function, such as ROS generation and arterial stiffness, has not already been extensively described. In this report, we present data from 18 males with high to very high CV risk undergoing LL treatment (LLT) with either statin and ezetimibe or ezetimibe monotherapy, who experienced, after a 2-month treatment with Evolocumab, a significant improvement in blood pressure (BP)-adjusted carotid–femoral pulse wave velocity (cfPWV) (*p*-value = 0.0005 in the whole cohort, *p*-value = 0.0046 in the sub-cohort undergoing background LLT with statin and ezetimibe, *p*-value = 0.015 in the sub-cohort undergoing background LLT with ezetimibe monotherapy), which was significantly associated with a decrease in freshly isolated leukocytes (PBMC_S_)-derived H_2_O_2_ production (*p*-value = 0.004, *p*-value = 0.02 and *p*-value = 0.05, respectively, in the whole cohort, in the statin + ezetimibe sub-cohort, and the ezetimibe sub-cohort). Our observations support the role of systemic oxidative stress in atherosclerosis and give a further rationale for using Evolocumab also for its effect in vascular disorders linked to oxidative processes.

## 1. Introduction

Reactive oxygen species (ROS) over-production and increased inflammation are involved in the cardiovascular (CV) functional and structural damage underlying CV disease (CVD) and its risk factors [1]. For example, oxidative stress increased in hypertension and increased ROS production has also been shown to promote atherosclerotic plaque formation [2,3].

Familial hypercholesterolemia (FH) is an autosomal dominant disorder characterized by high circulating levels of low-density lipoprotein cholesterol (LDL-C). Monogenic mutation in the LDL-receptor (LDL-R) gene may suppress protein synthesis, and consequently its translocation to the cell surface, reducing its turnover. Additional mechanisms include mutations affecting the apolipoprotein B (ApoB), the key structural component of LDL-C and the gene of pro-protein convertase subtilisin-Kexin type 9 (PCSK9), which promotes the LDL-R internalization and elimination in hepatocytes [4].

A high serum level of LDL-C is a recognized risk factor for the development of atherosclerosis-related CVD (ASCVD) [5], and LDL-C is highly susceptible to being modified by the oxidative milieu found inside the vascular wall. The “oxidative modification hypothesis of atherosclerosis” is based on the evidence that modified oxidized LDL-C is retained in atherosclerotic plaques, and its uptake by scavenger receptors on phagocytes leads to foam cell formation [6]. According to recent observations, FH has been associated with an increased generation of ROS, which is another key mechanism involved in atherosclerosis development and progression [7,8].

Evolocumab, a new lipid-lowering drug, is a fully human monoclonal antibody against PCSK9, which is able to prevent the hepatic LDL-R degradation induced by PCSK9, finally reducing the risk of ASCVD and limb events [9].

Treatment with Evolocumab has been shown to lower LDL-C levels and reduce the risk of CV events [10,11]. Recent reports have also suggested that the therapy with PCSK9 inhibitors (iPCSK9) promotes an improvement in arterial stiffness [12,13], even though the underlying molecular mechanism has not already been fully explained. Emerging evidence points out the role of PCSK9 as a vascular inflammation regulator by contributing to cytokines release, inflammatory cell recruitment, and atherosclerotic plaque formation [14]. In line with these observations, in vitro and in vivo experimental studies have shown that PCSK9 overexpression is positively related to an up-regulation of the TLR4 (Toll-like receptor 4)/NF-κB (nuclear factor-kappa B) proinflammatory pathway, thus contributing to vascular inflammation and endothelial dysfunction [15]. Moreover, recent experimental evidence underlines the contribution of PCSK9 also in the oxidative state within the arterial wall [16].

Interestingly, investigational results showed that Evolocumab significantly protects endothelial cells from H_2_O_2_-induced mortality by increasing antioxidant capacity and reducing hydroperoxides, malondialdehyde, and lipid peroxide levels [17].

According to these findings, further investigation of oxidative stress involvement may be the link in unveiling the pleiotropic effects of iPCSK9s in vascular homeostasis, beyond LDL-C lowering.

Based on the crucial role that ROS play within the CV system and more generally in CVD, monitoring ROS levels in humans would be important for both prognostic and diagnostic purposes (changes in oxidative stress level parallel the progression of the pathological condition) as well as to evaluate the response to treatment. In this regard, a major disadvantage is due to the limited availability of tissue samples from both heart and blood vessels. To overcome this problem, circulating markers of CV stress condition could be used. Notably, several studies performed over the last 15 years have identified circulating leukocytes as a suitable indicator of systemic CV stress conditions requiring minimal invasive intervention [18].

Peripheral blood mononuclear cells (PBMCs) (i.e., blood lymphocytes and monocytes) are one of the primary contributors to systemic ROS, playing a key role not only in the immune system but also in the inflammation state (cytokines production), endothelial dysfunction (endothelium adhesion molecule production) and CVD [19,20]. Interestingly, reports consistently demonstrate a reduced mitochondrial respiratory chain oxidative capacity related to the degree of CVD severity and an increased ROS production by PBMCs [21,22], which is possibly due to other pro-oxidant pathways such as the nicotinamide adenine dinucleotide phosphate (NADPH) oxidase enzyme family (NOXs) [23] activated by different stimuli (i.e., cytokines, growth factors, angiotensin II and oxLDL) [24]. Atherosclerosis is a systemic disease, and understanding the implication of circulating PBMCs and ROS production in patients with cardiac impairments appears worthwhile.

Below, we present data from 18 male subjects with high or very high CV risk who experienced 2-month treatment with the iPCSK9 Evolocumab. Since PBMCs can be considered one of the main sources of ROS in the vasculature, we tested the hypothesis that higher H_2_O_2_ production in PBMCs is associated with variable improvements in arterial stiffness depending on patients’ background lipid-lowering treatment.

## 2. Materials and Methods

### 2.1. Chemicals

The dioxetane CL probe (AquaSpark™ Peroxide Probe) was kindly provided by Biosynth (Staad, Switzerland). A 10 mM stock solution was prepared by dissolving the probe in DMSO (the CL probe solution is stable for months when stored at 4 °C and protected from light).

Phosphate-buffered saline (PBS) tabs (giving a 137 mM NaCl, 2.7 mM KCl and 10 mM phosphate buffer solution, pH 7.4), H_2_O_2_ solution, and pro-oxidant agent phorbol myristate acetate (PMA) were purchased from Sigma-Aldrich (St Louis, MO, USA). 

Ficoll-Paque™ PLUS was purchased from GE Healthcare (Boston, MA, USA).

Dulbecco’s Modified Eagle Medium (DMEM) high-glucose and MEM Non-Essential Amino Acids solution 100× were purchased from Microgem (Naples, Italy). Antibiotic solution 100× (10,000 U/mL penicillin and 10 mg/mL streptomycin) was purchased from Sigma-Aldrich. 

A Cytotoxicity LDH Assay kit was purchased from Dojindo Molecular Technologies (Rockville, MD, USA).

All other chemicals and solvents were of the highest analytical grade. 

Stock solutions of the pro-oxidant phorbol myristate acetate (PMA, 10 mM) were prepared by dissolving the compounds in DMSO.

### 2.2. Study Population

This is a sub-analysis of a main observational study, whose protocol was approved by the Ethics Committee of the University of Bologna.

**Figure 1:**
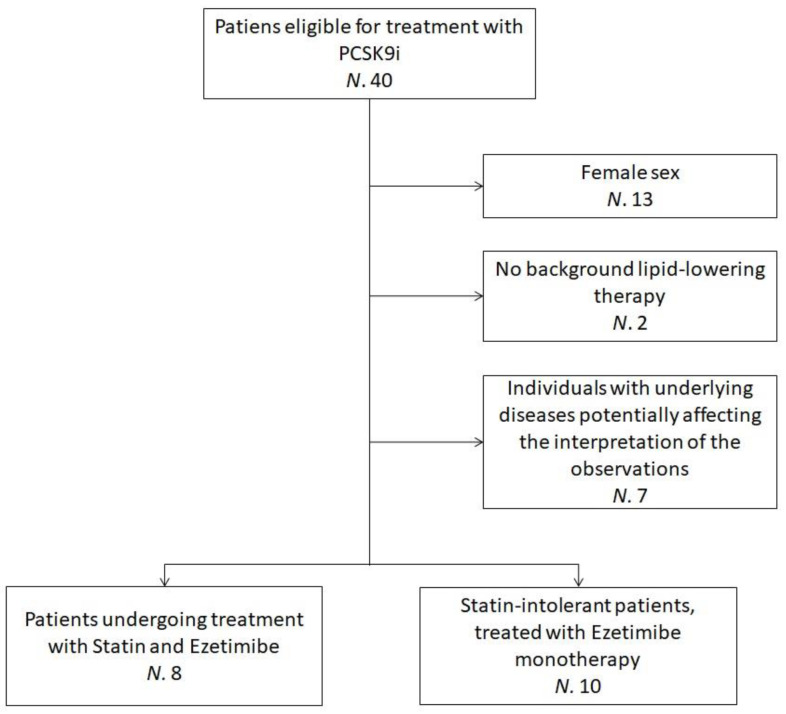
Flowchart detailing the recruitment process—the flowchart refers to patients clinically evaluated between March and November 2019. *N* = Number of individuals; PCSK9i = Protein convertase subtilisin/kexin type 9 inhibitors.

The sub-analysis pooled data from 18 hypercholesterolemic volunteers who were recruited at the Lipid Clinic of the S. Orsola-Malpighi University Hospital, Bologna, Italy. Enrolled subjects were eligible for treatment with PCSK9 inhibitor (iPCSK9) in agreement with the European Society of Cardiology (ESC)/European Atherosclerosis Society (EAS) clinical guidance recommendations [25] and the criteria released by the Italian regulatory agency AIFA [26,27]. Additional inclusion criteria for this sub-analysis were ≥18 years of age, male sex and being on oral lipid-lowering therapy (statin and ezetimibe or ezetimibe monotherapy) for ≥6 months before starting Evolocumab, with no planned dose change. Individuals with underlying diseases potentially affecting the interpretation of the study’s observations were excluded (Figure 1).

All individuals signed an informed consent to participate. The study followed the Declaration of Helsinki and its amendments.

### 2.3. Treatment

Patients were evaluated anamnestically and by the execution of physical examination, non-invasive vascular tests, and laboratory analyses before and after two months of treatment with 140 mg Evolocumab every 2 weeks. All laboratory and instrumental measurements were carried out by trained staff who followed standardized protocols.

### 2.4. Assessments

#### 2.4.1. Clinical Assessments

Patients’ personal history was evaluated paying particular attention to ASCVD, smoking habits, and ongoing pharmacological treatments. FH was diagnosed genetically. Height and weight were measured by standard procedures [28].

#### 2.4.2. Biochemical Analyses

Biochemical analyses were carried out on venous blood withdrawn from the basilic vein after at least 12 h of fasting. Plasma was obtained by the addition of disodium ethylenediaminetetraacetate (Na_2_EDTA) (1 mg/mL) and blood centrifugation at 3000 RPM for 15 min at room temperature.

Immediately after centrifugation, laboratory analyses were performed by standardized methods by trained personnel [29] to assess total cholesterol (TC), high-density lipoprotein-cholesterol (HDL-C), triglycerides (TG), lipoprotein(a) (Lp(a)), apolipoprotein B (apoB), fasting glucose (FPG), serum uric acid (SUA), creatinine (Cr), estimated glomerular filtration rate (eGFR), total and fractionated bilirubin, alanine transaminase (ALT), aspartate transaminase (AST), creatinine phosphokinase (CPK) and thyroid-stimulating hormone (TSH). LDL-C was calculated by the Friedewald formula.

#### 2.4.3. Blood Pressure Measurements

Systolic and diastolic blood pressure (SBP and DBP) measurements were performed using a validated oscillometric device in individuals at rest in the supine position, with a cuff of the appropriate size applied on the right upper arm. To improve detection accuracy, three BP readings were sequentially obtained at 3-min intervals. The first one was discarded, and the average between the second and the third was recorded [30].

#### 2.4.4. Non-Invasive Vascular Tests

Carotid-femoral pulse wave velocity (cfPWV) and ankle-brachial index (ABI) were non-invasively evaluated by the Vicorder^®^ apparatus (Skidmore Medical Ltd., Bristol, UK), which is a validated cuff-based oscillometric device.

CfPWV consists of the assessment of the pulse wave transmission through the arteries and is considered a reliable and early marker of arterial stiffness and a predictor of CV risk [31]. The theoretical basis of PWV is explained with the equation of Moens–Korteweg [32], and it is calculated as the length between two measurement sites divided by the time the pulse wave needs to cover that distance (m/s) [33]. During this study, cfPWV was calculated with the simultaneous measurement of carotid and femoral BP. A neck pad with a photoplethysmographic detector was placed around the neck, and a cuff was positioned around the patient’s thigh. The distance between the suprasternal notch and the thigh cuff represented the distance covered by the pulse wave in its carotid–femoral path and has been used by the Vicorder^®^ apparatus to establish the cfPWV value [34].

ABI measurement followed the American Heart Association (AHA) guidelines and the ESC/European Society of Hypertension (ESH) guidelines [30,35] and was assessed using Vicorder^®^ on the right and left sides, standing the patients supine. Hokanson SC10 cuffs were positioned on the upper arms and lower legs (above the ankles), and photoplethysmography sensors were clipped bilaterally to the end of the middle finger and the big toe. The cuffs were inflated up to 180 mmHg occluding the brachial and tibial arteries simultaneously. BP was taken at the point of the pulse returning at both sites as the cuffs slowly deflated.

Following the relevant International guidelines, ABI was calculated in each leg dividing the highest pressure between the posterior tibial and dorsalis pedis arteries by the highest arm pressure. The lowest ABI among the legs was considered as a study variable [36].

### 2.5. H_2_O_2_Bioassay Procedure

#### 2.5.1. PBMCs Collection

Fresh PBMCs were isolated from whole blood by Ficoll-Paque density gradient centrifugation [37] and resuspended in DMEM high glucose containing 10% FBS to a target concentration of 2 × 106 cells mL^−1^. The protocol foresees different steps: the whole blood (about 20 mL) was diluted 1:2 with PBS, and then about 40 mL of diluted blood was gently layered over 15 mL of Ficoll–Paque medium. Gradients were centrifuged at 400×  *g* for 30 min at 20°C in a swinging-bucket rotor without the brake applied. The PBMCs interface was carefully removed by pipetting, after which the PBMCs were washed with 50 mL of PBS and centrifuged at 200×g for 15 min removing the supernatant. This step was repeated twice. Finally, PBMC pellets were suspended in DMEM using 25 cm^2^ cell culture flasks and incubated at 37°C in a humidified atmosphere of 5% CO_2_. Cell counts and viability were assessed by Countess Automated Cell Counter (Thermo Fisher Scientific, Waltham, MA, USA).

#### 2.5.2. Quantification of Intracellular H_2_O_2_ in PBMCs

The selective CL probe for H_2_O_2_ designed by Shabat’s research group [38] was based on previous Schaap’s adamantylidene−dioxetane probes [39]. The working solutions of the CL probe (20 μM) and the pro-oxidant PMA (100 μM) were prepared by diluting the respective stock solutions with PBS.

One hundred microliters of suspension of freshly isolated PBMCs were plated in the wells of a 96-well black microtiter plate (2.0 × 10^5^–0.25 × 10^5^ cells well-1) and incubated for 20 min with 50 μL of the dioxetane CL probe working solution. Then, 50 μL of working solution of the pro-oxidant PMA (25 µM final concentration in well) was added to induce intracellular H_2_O_2_ production, and the CL emission was monitored for 60 min using a Luminoskan™ Ascent luminometric plate reader (PBS was used as the negative control).The whole assay was conducted at 37 °C. The integrated CL emission in the time interval between 40 and 60 min upon the addition of PMA was used as the CL analytical signal. The experimental results were analyzed by plotting the CL analytical signal versus the PBMC concentration and fitting the experimental data to a straight line using the method of least squares. The slope of the line was taken as an index of H_2_O_2_ production by PBMCs under the pro-oxidant stimulus.

### 2.6. Cell Viability Assay

The cell viability was assessed by WST8 (2-(2-methoxy-4-nitrophenyl)-3-(4-nitrophenyl)-5-(2,4-disulfophenyl)-2H-tetrazolium, monosodium salt) (Dojindo Molecular Technologies, Kumamoto, Japan) that in the presence of an electron mediator is reduced by dehydrogenases in cells (as a vitality biomarker) to formazan dye, which is soluble in the tissue culture medium. The amount of the formazan dye generated by dehydrogenases in cells is directly proportional to the number of living cells [40]. The absorbance signal between the beginning and the end of each experiment was monitored at 37 °C at 450 nm using a Varioskan™ Flash Multimode Reader.

### 2.7. Data Analysis

Baseline characteristics of each patient were reported as percentage frequencies for categorical variables and mean ± standard deviation (SD) for continuous variables. The normality distribution of the variables was tested using the D’agostino–Pearson omnibus normality test. Paired-sample T-tests (preceded by Levene’s test) and 1-way analysis of variances (ANOVA) were used to compare values obtained before and after treatment. Multiple variable analyses were carried out by analyzing the constructed correlation matrix using Pearson’s coefficient with blood-pressure adjusted cfPWV as the dependent variable, and H_2_O_2_, age, BMI, SUA, eGFR, and pre–post treatment variation in LDL-C as potential predictors. Then, a stepwise univariate correlation analysis was carried out between blood-pressure adjusted cfPWV and H_2_O_2_ in both study sub-cohorts. The correlation between two variables was analyzed as a function of the *p*-value (two-tailed *p*-values < 0.05 were always regarded as statistically significant). Furthermore, a correlation coefficient ≥ 0.7 was considered as the threshold for determining a strong correlation, while a correlation coefficient ≥ 0.4 means at least a moderate correlation.

Data were analyzed using GraphPad Prism v. 6.05 (GraphPad Software, Inc., La Jolla, CA, USA) was used to plot the experimental data and for the least-squares fitting of CL signal/PBMC concentration graphs.

## 3. Results and Discussion

Patients started treatment with Evolocumab between March 2019 and November 2019. In the overall study population, the mean age was 65.7 years. Demographic and clinical characteristics of each patient at baseline were reported in Table 1. Heterozygous FH (HeFH) affected 53.3% of patients, and pre-existing CVD and/or peripheral obliterative arterial disease were present in 60%. A total of 60% of patients had a story of statin intolerance and 26.7% were receiving high-dose statin treatment (20 or 40 mg of rosuvastatin per day). The mean baseline LDL-C level was 154.49 ± 67.84 mg/dL.

All patients in the intention-to-treat (ITT) population completed the 2-month follow-up.

### 3.1. Evolocumab Treatment Decreases Intracellular H_2_O_2_Production in PBMCs

For the first primary endpoint, a significant decrease in H_2_O_2_ production by PBMCs from baseline to 2-month follow-up was observed (*p*-value = 0.02).

Thanks to a recently developed rapid and selective bioassay able to quantify a very small intracellular amount of H_2_O_2_ in human living cells [41], we monitored the intracellular production of H_2_O_2_ in freshly isolated PBMCS. The bioassay employed an adamantylidene-1,2-dioxetane lipophilic probe containing an arylboronate moiety, which upon reaction with H_2_O_2_ is converted to the correspondent phenol, leading to probe decomposition and the formation of an excited-state fragment that emits light.

**Figure 2:**
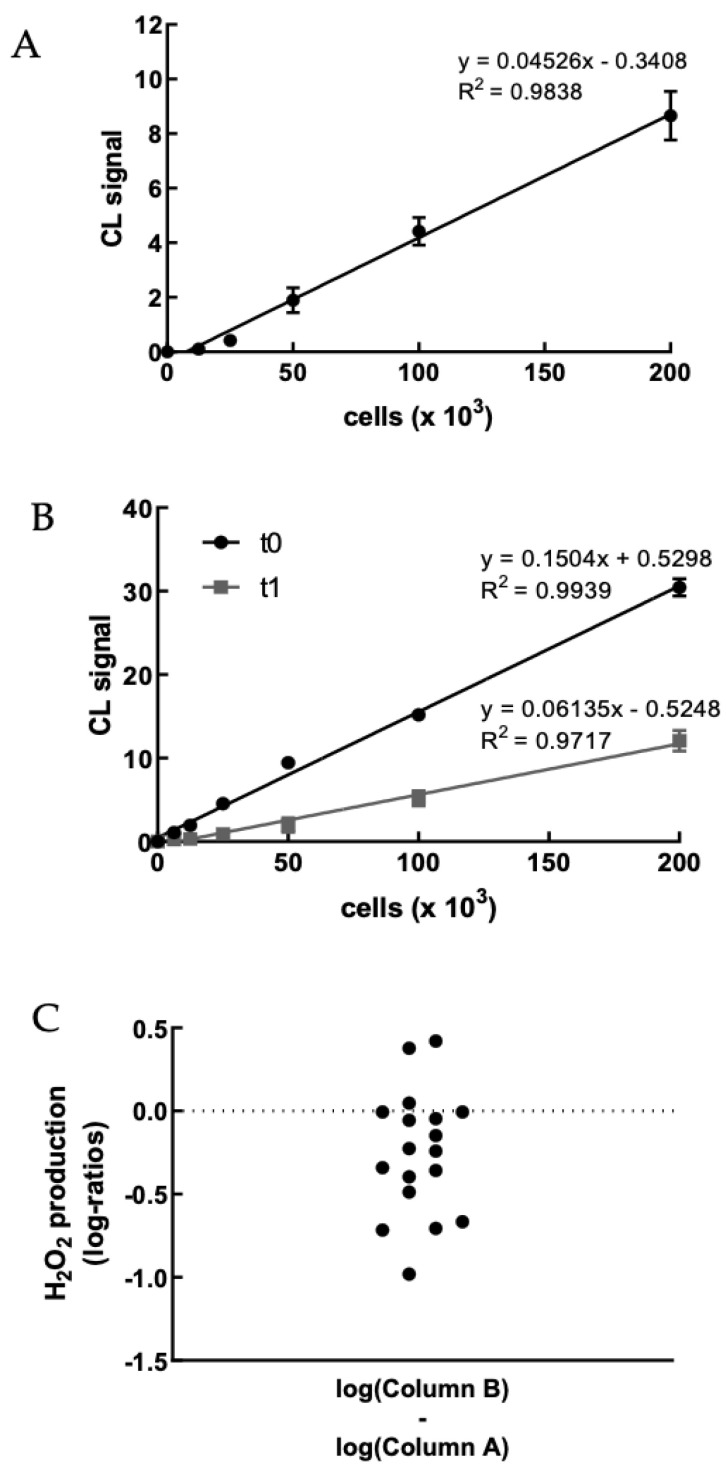

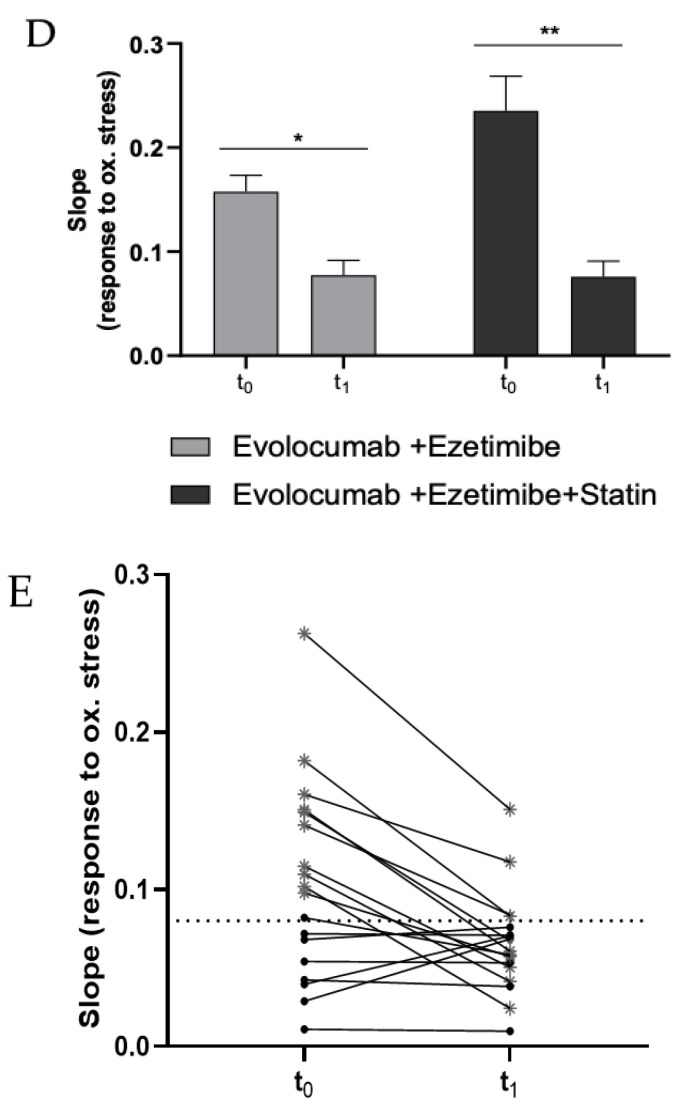
(**A**) Dose–response graph showing the correlation between the CL signal and the concentration of freshly isolated PBMCs from a healthy volunteer (range 0–2 × 10^5^ cells). Each point represents the mean ± SD of three independent measurements. (**B**) Dose–response graph showing the correlation between the CL signal and the concentration of freshly isolated PBMCs from a hypercholesterolemic subject before (t_0_) and after (t_1_) two-month treatment with Evolocumab. Each point represents the mean ± SD of three independent measurements. (**C**) Changes of H_2_O_2_ production (as log-ratios) by PBMCs isolated from 18 hypercholesterolemic subjects observed after two-month treatment with Evolocumab (H_2_O_2_ production was evaluated as the slope of the CL signal versus PBMCs concentration graphs). (**D**) Slope values before and after Evolocumab treatment (the slope was calculated from the calibration curve obtained correlating the CL signal versus PBMCs concentration pre- and post-treatment). * *p* ˂ 0.05, ** *p* ˂ 0.01 significantly different from t_0_ values. (**E**) Changes of H_2_O_2_ production by PBMCs isolated from 18 hypercholesterolemic subjects observed before (t_0_) and after (t_1_) two-month treatment with Evolocumab (H_2_O_2_ production is expressed as the slope value of each curve derived by CL signal versus PBMCs concentration graphs).

We first evaluated the H_2_O_2_ production of PBMCs obtained from healthy volunteer staff members upon treatment with PMA to set up the method (Figures S1 and S2). Figure 2A shows a representative example of the correlation between CL signal and PBMC concentration. As expected, the CL emission intensity linearly increased with the concentration of cells up to 2 × 10^5^ cells well^−1^. We were particularly interested in the possibility of sensitively detecting the H_2_O_2_ produced by a small number of PBMCsfor being able to perform the assay using painless not-invasive blood sampling tools such as HemoLink shortly [42].

Next, the dioxetane CL probe was employed to compare the intracellular production of H_2_O_2_ in freshly isolated PBMCs from 18 hypercholesterolemic subjects before (t_0_) and after two-month treatment with Evolocumab (t_1_). As an example, Figure 2B shows a statistically significant decrease (*p*-value < 0.0001) in the slope of the CL signal vs. PBMCs concentration graph obtained using PBMCs isolated from enrolled subjects at t_0_ and t_1_, indicating that H_2_O_2_ production from PBMCs decreased after Evolocumab treatment. When considering all the subjects enrolled in the study, it can be observed that despite the small number of patients (*n* = 18), a ratio paired-sample *t*-test indicated that treatment with Evolocumab significantly reduced the production of H_2_O_2_ from PBMCs (*p*-value = 0.02), suggesting a possible beneficial effect in endothelial function (Figure 2C). Notably, we monitored PBMCs viability before and after each experiment, obtaining no significant differences (data not shown), thus confirming that Evolocumab treatment significantly reduces H_2_O_2_ production from PBMCs. Interestingly, we compared slope values of all the 18 patients pre and after Evolocumab treatment, observing in both sub-cohorts a significant tendency to decrease (Ezetimibe group, *p*-value = 0.04; Ezetimibe + statin group, *p*-value = 0.009), which was mainly when the slope value is higher than the 0.08 threshold (Figure 2D,E).

The numerous efforts made over the last decades to develop tools able to monitor the oxidative stress level in patients affected by CVD rely on the need to gain information on the disease state. Among others, the isolation of circulating leukocytes to measure the oxidant level offers a valid, non-invasive challenge that has been tested in a few pathological contexts, including hypertension, atherosclerosis and its clinical manifestations, and heart failure. Since leukocytes circulate in the bloodstream, it is expected that they might reflect quite closely both systemic and cardiovascular oxidative stress and provide useful information on the pathological condition [43]. Moreover, the measurement of leukocyte oxidant activities may reveal the importance to monitor the effectiveness of specific therapies, such as antihypertensive drugs [44]. Several studies support the concept that dysfunctional circulating PBMCs can act as an amplifier of oxidative stress and cell/tissue damage, ultimately contributing to the progression of the HF condition and atherosclerosis [45,46]. Consequently, it is not surprising that the PBMCs-derived ROS level predicts hospital readmission in chronic HF [47].

A possible limitation linked with the analysis of PBMCs derived ROS consists of the inter and intra-individual biological variability of H_2_O_2_ production. In this study, several strategies were adopted to overcome this limitation. The sampling was performed at the same time in the early morning for all patients before and after the treatment, since it is well-known that there is a large daily fluctuation in ROS production [48].

Moreover, all the patients were males, over 18 years, and without diseases and relative treatment, which could potentially affect the oxidative state. The experimental design, which includes the ratio between post and pre-treatment values in every single subject, and the three-time repetition of measurements minimizes the H_2_O_2_ production variability within individual experiments.

### 3.2. Plasma Lipids and Arterial Stiffness Improvement after Treatment with Evolocumab

As expected, patients in both sub-cohorts experienced significant improvements in serum lipid levels after treatment with PCSK9 inhibitor Evolocumab (Table 2).

**Figure 3:**
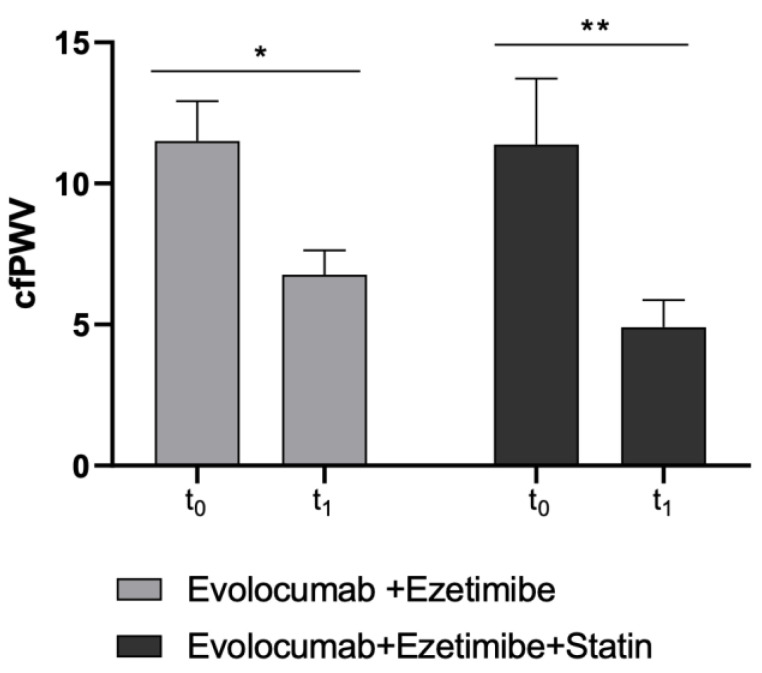
Changes in cfPWV values before and after Evolocumab treatment in study sub-cohorts (Ezetimibe group and Ezetimibe+ Statin group). * *p* < 0.05 and ** *p* < 0.01 significantly different vs. t_0_ values.

Moreover, as the second primary endpoint, cfPWV significantly improved in both study sub-cohorts (Ezetimibe + Statin group: *p*-value = 0.0046; Ezetimibe group: *p*-value = 0.015) (Table 3 and Figure 3).

cfPWV is a benchmark clinical measure of large elastic arterial stiffness and was found to be a powerful predictor of CVD in the population. It is now well established that increased vascular stiffness signifies a declining in vascular function and an associated increased CV risk. In this context, changes in the arterial wall, including increased collagen deposition and degradation of elastin, have already been associated with changes in the mechanical properties of the arteries. Furthermore, data from a recent placebo-controlled randomized clinical study found that the combination of statin and Evolocumab produces favorable changes in coronary atherosclerosis consistent with plaque stabilization and regression [49].

### 3.3. H_2_O_2_ Decrease Correlates with Arterial Stiffness Improvement

In the multiple correlation model, changes in 2-month BP-adjusted cfPWV were significantly associated with changes in H_2_O_2_ (Pearson coefficient R = 0.65, *p*-value = 0.004). However, cfPWV did not correlate either with age, BMI, eGFR, and SUA levels at baseline or with the percentage change in LDL-C at the 2-month follow-up (*p*-values > 0.05 for all the considered variables). In line with our data, Maulucci and co-workers previously showed that a 2-month treatment with 140 mg Evolocumab improves endothelial function in subjects with increased CV risk, and this improvement correlated to LDL-C reduction [50]. In addition, it has recently been reported that subjects treated with iPCSK9 experienced a reduction in cfPWV even when LDL-C increases, suggesting that the iPCSK9 treatment could independently exert beneficial effects on vasomotor function [12]. This may contribute to CVD risk reduction independently of changes in serum lipoproteins concentration [51].

In our samples, the improvement in cfPWV in both sub-cohorts correlates with the reduction in H_2_O_2_ production (R = 0.77 *p*-value = 0.02 and R = 0.56 *p*-value = 0.05, respectively in Statin + Ezetimibe and Ezetimibe monotherapy sub-cohorts). Indeed, a large body of evidence supports that chronic low-level inflammation may have an important role in atherosclerosis, being an early event in the pathology of ASCVD rather than a consequence [52]. Persistent low-grade inflammation is associated with the increase in systemic levels of proinflammatory cytokines that in turn favors PBMCs being in a state of preactivation against an injury [53].

In the past, the short-term effect of statins on arterial stiffness was attributed at least in part to their antioxidant and anti-inflammatory properties [54]. In this study, the stronger correlation between cfPWV decrease and H_2_O_2_ production in the Ezetimibe + Statin sub-cohort rather than Ezetimibe group could be linked to the combination of iPCSK9 and statin treatment. In summary, our results suggest that the beneficial effect on arterial stiffness could be attributed to LDL-C change that, in turn, promotes a decreased vascular oxidative stress.

## 4. Conclusions

In conclusion, cfPWV and PBMCs-derived H_2_O_2_ production profiles in hypercholesterolemic individuals significantly improved after short-term treatment with the iPCSK9 Evolocumab. Our results appear to be consistent with the beneficial role of this novel injectable lipid-lowering therapy. Of course, some limitations need to be addressed. The main one is the small sample size, which however was justified by the exploratory nature of the analysis carried out in a selected cohort of a specific kind of subjects. Furthermore, other parameters regarding the evaluation of CV risk in hypercholesterolemic subjects—including oxidized LDL-C—were not available and were not taken into consideration. Finally, we did not consider either indicator of lipid peroxidation or any marker of vascular oxidative stress other than H_2_O_2_.

For these reasons, these preliminary findings should be further confirmed in an adequately powered long-term placebo-controlled randomized clinical trial to evaluate the effect of iPCSK9 on arterial stiffness and oxidative stress in a selected clinical setting through proper statistical means. Nevertheless, we were able to show significant improvements in cfPWV and PBMCs-derivedH_2_O_2_ production profile after Evolocumab treatment in both Statin + Ezetimibe and Ezetimibe study cohorts.

A genomic fingerprint of PBMCs derived from high CV-risk subjects will be also assessed shortly to better characterize the effect of Evolocumab treatment in the gene expression profile differences associated with the health status of a human subject.

## Figures and Tables

**Table 1:** Baseline clinical characteristics of the cohort.

Characteristics	Statin and Ezetimibe (*N* = 8)	Ezetimibe (*N* = 10)
Age (years)—mean ± SD	63.2 ± 7.3	69.4 ± 4.9
Active smokers—%	37.5	10
Heterozygous FH—%	62.5	30
Primary cardiovascular prevention—%	25	40
Secondary cardiovascular prevention—%	75	60
Cardiovascular disease—%	75	40
Cerebrovascular disease—%	0	10
Peripheral obliterative arterial disease—%	12.5	20
Type 2 diabetes—%	0	20
Hypertension—%	50	70
History of statin intolerance—%	50	80
Low-dose * statin treatment—%	50	0
High-dose ^§^ statin treatment—%	50	0
n-3 PUFA treatment—%	25	0
Urate-lowering treatment—%	12.5	10

* defined as 5 or 10 mg Rosuvastatin; ^§^ defined as 20 or 40 mg Rosuvastatin. FH = Familial hypercholesterolemia; *N* = Number of individuals; PUFA = Poly-unsaturated fatty acids; SD = Standard deviation.

**Table 2:** Laboratory data of the patients undergoing treatment with Evolocumab.

Parameters	Statin and Ezetimibe (*N* = 8)		Ezetimibe (*N* = 10)	
	Baseline (Mean ± SD)	Follow-Up (Mean ± SD)	Baseline (Mean ± SD)	Follow-Up (Mean ± SD)
Fasting plasma glucose—mg/dL	94.13 ± 5.08	93.4 ± 8	93.6 ± 10.04	99.6 ± 14.48
Creatinine—mg/dL	0.81 ± 0.13	0.78 ± 0.15	0.95 ± 0.16	1 ± 0.18
eGFR—mL/min/1.73 m^2^	89.16 ± 17.11	90.63 ± 18.52	73.78 ± 15.34	68.11 ± 16.09
Serum uric acid—mg/dL	5.05 ± 0.93	4.61 ± 0.85 *	5.86 ± 0.91	6 ± 0.98
Total cholesterol—mg/dL	212.88 ± 35.92	111.75 ± 12.15 *	247.4 ± 85.19	138.3 ± 60.73 *
Triglyceride—mg/dL	137.75 ± 93.23	106.5 ± 64.36 *	138.7 ± 66.67	153 ± 150.69
HDL-cholesterol—mg/dL	58 ± 13.22	58 ± 11.87	56.2 ± 12.47	55.2 ± 13.58
Non-HDL-cholesterol—mg/dL	154.88 ± 32.99	53.75 ± 8.88 *	191.2 ± 78.63	83.1 ± 52.84 *
LDL-cholesterol—mg/dL	132.54 ± 35.94	33.4 ± 12.92 *	163.46 ± 78.16	52.5 ± 55.33 *
VLDL-cholesterol—mg/dL	28.03 ± 20.09	21.31 ± 13.9 *	28.16 ± 14.07	32.07 ± 31.59
Apolipoprotein-B—mg/dL	147.2 ± 47.8	53 ± 20.48 *	148 ± 41.12	53.63 ± 33.62 *
Lipoprotein(a)—mg/dL	95.72 ± 92.62	67.06 ± 58.42	42.05 ± 54.45	30.79 ± 42.15 *
Total bilirubin—mg/dL	0.5 ± 0.07	0.54 ± 0.12	0.64 ± 0.18	0.58 ± 0.21
Direct bilirubin—mg/dL	0.13 ± 0.04	0.16 ± 0.09	0.11 ± 0.03	0.13 ± 0.05 *
Indirect bilirubin—mg/dL	0.37 ± 0.05	0.37 ± 0.06	0.54 ± 0.15	0.46 ± 0.17
Aspartate aminotransferase—U/L	25.71 ± 7.7	26.71 ± 8.73	33.2 ± 24.79	31.9 ± 26.59
Alanine aminotransferase—U/L	24.13 ± 10.26	23.5 ± 9.93	27.4 ± 14.97	24.6 ± 16.15
Gamma-glutamyl transferase—U/L	36 ± 51.8	45.13 ± 82.16	32.5 ± 23.63	29.8 ± 13.74
Creatine phosphokinase—U/L	125.25 ± 78.04	150.38 ± 95.58	393.8 ± 392.8	361.2 ± 398.3
Thyroid-stimulating hormone—μU/mL	1.92 ± 1.09	2.06 ± 1.2	2.44 ± 0.77	2.03 ± 0.38

eGFR = Estimated glomerular filtration rate; HDL = High-density lipoprotein; LDL = Low-density lipoprotein; *N* = Number of individuals; SD = Standard deviation; VLDL = Very-low density lipoprotein. * *p* < 0.05 vs. baseline.

**Table 3:** Pre-treatment and 2-month follow-up hemodynamical data of enrolled patients.

Parameters	Statin and Ezetimibe (*N* = 8)		Ezetimibe (*N* = 10)	
	Baseline (Mean ± SD)	Follow-Up (Mean ± SD)	Baseline (Mean ± SD)	Follow-Up (Mean ± SD)
Heart rate—bpm	67.13 ± 13	68.75 ± 8.19	62.8 ± 7.18	64.1 ± 10.04
Systolic blood pressure—mmHg	135.13 ± 14.29	148.13 ± 17.76 *	142.3 ± 20.87	153 ± 14.31
Diastolic blood pressure—mmHg	70.5 ± 10.3	71.1 ± 10.5	70.1 ± 11.1	75.4 ± 8.1
Pulse wave velocity—m/s	11.39 ± 1.26	4.91 ± 2.7 **	11.5 ± 1.9	6.77 ± 2.71 *
Ankle–brachial index (right)	1.06 ± 0.06	1.1 ± 0.15	0.91 ± 0.23	1.08 ± 0.07
Ankle–brachial index (left)	1.02 ± 0.18	1.06 ± 0.21	0.98 ± 0.19	1.1 ± 0.2

*N* = Number of individuals; SD = Standard deviation. * *p* < 0.05 versus baseline; ** *p* < 0.01 vs. baseline.

## Data Availability

The data that support the findings of this study are available from the Corresponding Author with the permission of the University of Bologna.

## Supplementary Materials

The following supporting information can be downloaded at: https://www.mdpi.com/article/10.3390/antiox12030578/s1, Figure S1: (A) Dose-response graph showing the correlation between the CL signal and the concentration of PMA (range 0–25 µM) in freshly isolated PBMCs from a healthy volunteer (25–50–100 × 10^3^ cells). Each point represents the mean ± SD of three independent measurements. (B) Chemiluminescence kinetic profiles obtained for PBMCs isolated PBMCs from a healthy volunteer in the presence of different concentrations of PMA; Figure S2: Dose-response curves showing the correlation between the CL signal and the concentration of PMBCs from a healthy volunteer (range 0–200 × 10^3^ cells) in the presence of PMA (12.5 and 25 µM, respectively). Each point represents the mean ± SD of three independent measurements.
