# Supplementary material for: Treatment with PCSK9 Inhibitor Evolocumab Improves Vascular Oxidative Stress and Arterial Stiffness in Hypercholesterolemic Patients with High Cardiovascular Risk
Source: Antioxidants (Basel). 2023 Feb 25;12(3):578. doi: 10.3390/antiox12030578 (PMC10045769; doi:10.3390/antiox12030578)
Supplement: Supplementary file 1 [file antioxidants-12-00578-s001.zip › antioxidants-2207663-supplementary.pdf]

Supplementary Materials

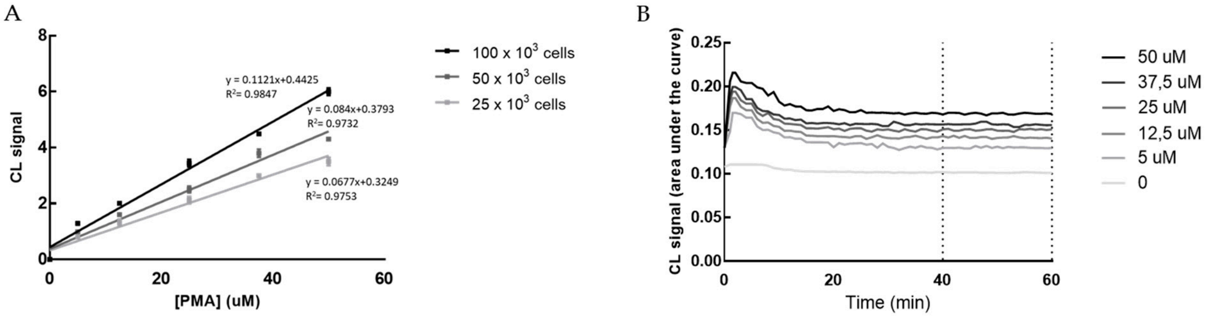

Figure S1.(A) Dose-response graph showing the correlation between the CL signal and the concentration of PMA (range 0 – 25 μM) in freshly isolated PBMCs from a healthy volunteer (25 – 50 – 100 x 10^3 cells). Each point represents the mean ± SD of three independent measurements. (B)Chemiluminescence kinetic profiles obtained for PBMCs isolated PBMCs from a healthy volunteer in the presence of different concentrations of PMA.

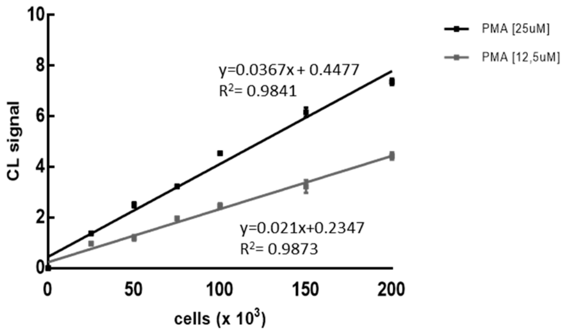

Figure S2. Dose-response curves showing the correlation between the CL signal and the concentration of PMBCs from a healthy volunteer (range 0 – 200 x 10^3 cells) in the presence of PMA (12.5 and 25 μM, respectively). Each point represents the mean ± SD of three independentmeasurements.
